# Sub-millisecond 2D MRI of the vocal fold oscillation using single-point imaging with rapid encoding

**DOI:** 10.1007/s10334-021-00959-4

**Published:** 2021-09-20

**Authors:** Johannes Fischer, Ali Caglar Özen, Serhat Ilbey, Louisa Traser, Matthias Echternach, Bernhard Richter, Michael Bock

**Affiliations:** 1 Department of Radiology, Medical Physics, University Medical Center Freiburg, Faculty of Medicine, University of Freiburg, Freiburg, Germany; 2 German Consortium for Translational Cancer Research Partner Site Freiburg, German Cancer Research Center (DKFZ), Heidelberg, Germany; 3 Freiburg Institute for Musicians’ Medicine, Freiburg University Medical Center, Faculty of Medicine, University of Freiburg, Freiburg, Germany; 4 Division of Phoniatrics and Pediatric Audiology, Department of Otorhinolaryngology, Head and Neck Surgery, Ludwig-Maximilians-University, Munich, Germany

**Keywords:** Magnetic resonance imaging, Dynamic MRI, Sub-millisecond MRI, Vocal chords, High temporal resolution MRI

## Abstract

**Objective:**

The slow spatial encoding of MRI has precluded its application to rapid physiologic motion in the past. The purpose of this study is to introduce a new fast acquisition method and to demonstrate feasibility of encoding rapid two-dimensional motion of human vocal folds with sub-millisecond resolution.

**Method:**

In our previous work, we achieved high temporal resolution by applying a rapidly switched phase encoding gradient along the direction of motion. In this work, we extend phase encoding to the second image direction by using single-point imaging with rapid encoding (SPIRE) to image the two-dimensional vocal fold oscillation in the coronal view. Image data were gated using electroglottography (EGG) and motion corrected. An iterative reconstruction with a total variation (TV) constraint was used and the sequence was also simulated using a motion phantom.

**Results:**

Dynamic images of the vocal folds during phonation at pitches of 150 and 165 Hz were acquired in two volunteers and the periodic motion of the vocal folds at a temporal resolution of about 600 µs was shown. The simulations emphasize the necessity of SPIRE for two-dimensional motion encoding.

**Discussion:**

SPIRE is a new MRI method to image rapidly oscillating structures and for the first time provides dynamic images of the vocal folds oscillations in the coronal plane.

**Supplementary Information:**

The online version contains supplementary material available at 10.1007/s10334-021-00959-4.

## Introduction

The vocal folds are a key component in the production of human voice. Driven by a subglottal pressure built up by expiratory forces, the vocal folds oscillate and produce sound (phonation) which is modulated by the vocal tract acoustics to allow for a range of expressions in speech and singing. During the latter, the vocal folds can oscillate with fundamental frequencies higher than 1500 Hz [1]. However, vocal fold pathologies including mass lesions [2, 3], Reinke’s edema [4, 5], sulcus vocalis [6] or cancer [7, 8] can significantly alter the mechanical properties of the vocal folds leading to hoarseness or even the inability to phonate. To determine the effect of such vocal fold mass lesions on the voice source production, imaging techniques are essential [9, 10]. In addition to static diagnostic imaging, dynamic visualization techniques of vocal fold motion can furthermore provide a better functional understanding of the phonation.

The vocal folds consist of the *musculus vocalis* and the *lamina propria*, a non-muscular layered structure containing elastin and collagen fibers, which is covered by a thin epithelial layer [11]. This oscillatory system can be described by the biomechanical cover-body model that assumes coupled oscillators of different masses in each vocal fold [12–14]. The closure of the vocal folds during phonation is performed by the surface layers in an upward and later side-traveling mucosal wave. In male singers, at a phonation frequency of 150 Hz, a mucosal wave velocity of 4 m/s can be expected [15]. To better understand the motion and the model, many visualization techniques have been applied.

Currently, laryngeal stroboscopy [16, 17] is the gold standard in dynamic vocal fold imaging—here, an endoscope is inserted transnasally or transorally with a view to the larynx including the vocal folds, and images are acquired in the presence of a stroboscopic illumination which is slightly detuned to the vibration frequency of the vocal folds to capture different phases of the oscillatory motion. Thus, laryngeal stroboscopy provides a two-dimensional top-down view of the oscillating vocal folds without any depth information. The same holds for high-speed glottography, where the temporal resolution is much higher than with stroboscopy [18]. Depth-kymography on the other hand allows to extract depth information from the vocal fold topology via laser triangulation [19], and optical coherence tomography (OCT) can differentiate individual layers within the vibrating vocal folds [20]. These imaging techniques share the limitation that the vocal folds can be examined only in a top-down view or need very close contact with the vocal folds. Cross-sectional measurements of the vocal fold velocities have been performed in the coronal plane using external ultrasonic transducers [21], or in excised hemilarynges [22, 23]. Compared to these imaging techniques, MRI is less invasive, it offers different soft tissue contrasts, and it has the potential to visualize the complex motion pattern in arbitrary slice orientations. MRI is already an established technique for the visualization of vocal tract dynamics in voice production [24–26] and singing [27–29]. Recently, dynamic 3D reconstructions of speech production with 15 fps have been presented [30].

To overcome the depth limitations of conventional imaging, we recently applied MRI to dynamically visualize the rapid oscillation of the vocal folds in the transverse plane [31]. Dynamic vocal fold MRI is challenging as the oscillation frequencies are on the order of or higher than the typical switching frequencies of the MRI gradients. Using fast gradient switching in phase-encoding (PE) direction and simultaneous electrophysiologic measurements [32], we could resolve the 1D motion of the vocal folds in left–right (LR) direction with sub-millisecond temporal resolution. Dynamic images in transverse orientation provide similar information than conventional imaging techniques—clinically, a coronal cross-sectional view would be desirable as it could demonstrate both the LR closure and the propagating mucosal wave in inferior–superior direction.

In this work, we propose a 2D single-point imaging with rapid encoding (SPIRE) technique for dynamic imaging, where the temporal resolution does not depend on TR but on the much shorter duration of time-optimized PE gradients applied in both in-plane directions.

The pulse sequence is based on the single-point imaging (SPI) technique [33], which uses only PE gradients for spatial encoding. Thus, only a single k-space point is acquired per RF excitation which makes the acquisition time proportional to the number of k-space points. SPI ensures a constant echo time (TE) for all points in k-space which avoids k-space filtering from T_2_* decay for materials with ultrashort T_2_* [34], so that SPI is the preferred imaging method for applications in porous media [35, 36] and solid materials [37, 38]. The long acquisition times of SPI can be shortened by applying the excitation pulse already in the presence of ramped up encoding gradients [39]. SPI is also applied in hybrid zero echo time sequences to acquire data close to k-space center [40, 41].

## Methods

### SPIRE pulse sequence

**Fig. 1:**
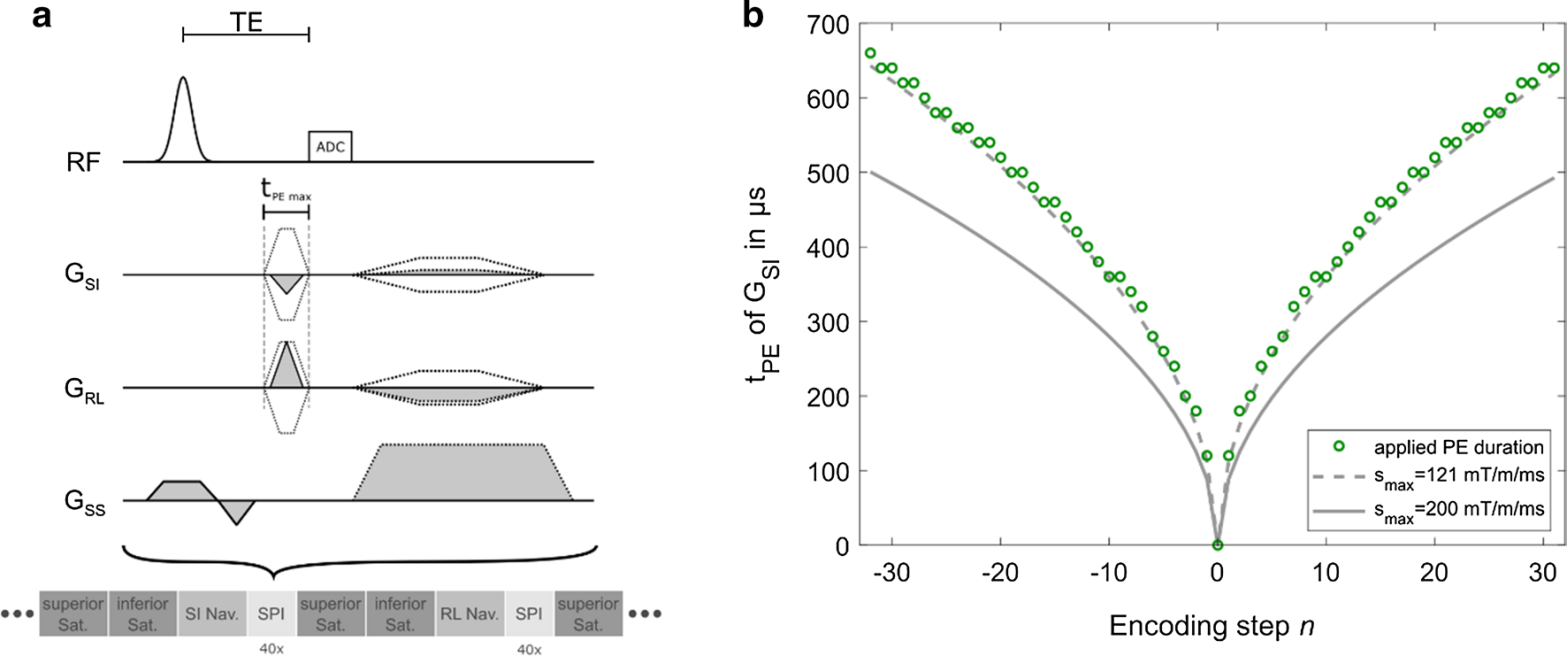
**a** Sequence diagram of the proposed SPIRE method. PE occurs in both in-plane directions, and PE gradients are applied for the shortest possible time. Dotted lines indicate the gradient shapes for the largest phase encoding moments. The two saturation bands are excited, followed by the acquisition of an SI or RL projection used as a navigator. Navigators are followed by 40 SPI acquisitions, where the ADC follows immediately after the rapidly switched phase encoding gradients. **b** Duration of *G*_SI_ depending on the encoding step *n*. In our measurements, the gradient shapes remain triangular for all k-space points. The steps in the gradient duration stem from the 10 µs raster of the gradient system. Gray lines show the achievable duration with a given slew rate

The SPIRE sequence is an extension of our 1D-motion encoding method [31]. Here, time-optimized PE was performed along the LR direction in which the fast vocal fold motion occurs. The duration of the PE gradients is shorter than the oscillatory motion displacement of the vocal folds, so that quasi-static images of the vocal folds can be reconstructed for different phases of the oscillation. In anterior–posterior direction, conventional longer frequency encoding gradients were used, making this technique unsuitable for rapid 2D motion encoding. Thus, in SPIRE short PE gradients are applied in both in-plane directions and no frequency-encoding gradient is used during data acquisition (Fig. 1a).

The sequence was implemented on a clinical 3 T MRI system (PrismaFit, Siemens, Germany) with a maximum gradient amplitude of *G*_max_ = 80 mT/m and a maximum slew rate of *s*_max_ = 200 mT/m/ms. PE durations, *t*_PE_, were minimized by using triangular gradient shapes with high slew rates. The duration of the *n-*th PE gradient is given by

1 $${t}_{\mathrm{PE}}\left(n\right)=2\cdot \sqrt{\frac{n}{N\cdot \gamma \cdot \Delta x\cdot {s}_{\mathrm{max}}}}.$$

$N$ is the total number of PE steps in one image dimension, $\Delta x$ is the in-plane resolution, and $\gamma = \mathrm{42,577}\ \mathrm{MHz}/\mathrm{T}$ is the gyromagnetic ratio. With this gradient system and a spatial resolution of $\Delta x$ = 1 mm, the outermost k-space point can be encoded with a PE gradient of ${t}_{\mathrm{PE max}} =$ 485 µs duration (Fig. 1b). When the gradient amplitude ${G}_{\mathrm{PE}}(n)$ reaches the maximum realizable gradient strength ${G}_{\mathrm{max}}$, i.e., for

$$n\ge \frac{\gamma {{G}_{\mathrm{max}}}^{2}N\Delta x}{{s}_{\mathrm{max}}},$$

trapezoidal PE gradients are applied, and *t*_PE_ increases linearly with *n*. Data from a single k-space point are acquired for about 100 µs at a constant TE which is determined by ${t}_{\mathrm{PE max}}$. After the acquisition block, remaining transverse magnetization is spoiled along slice direction and PE gradients are rewound.

**Fig. 2:**
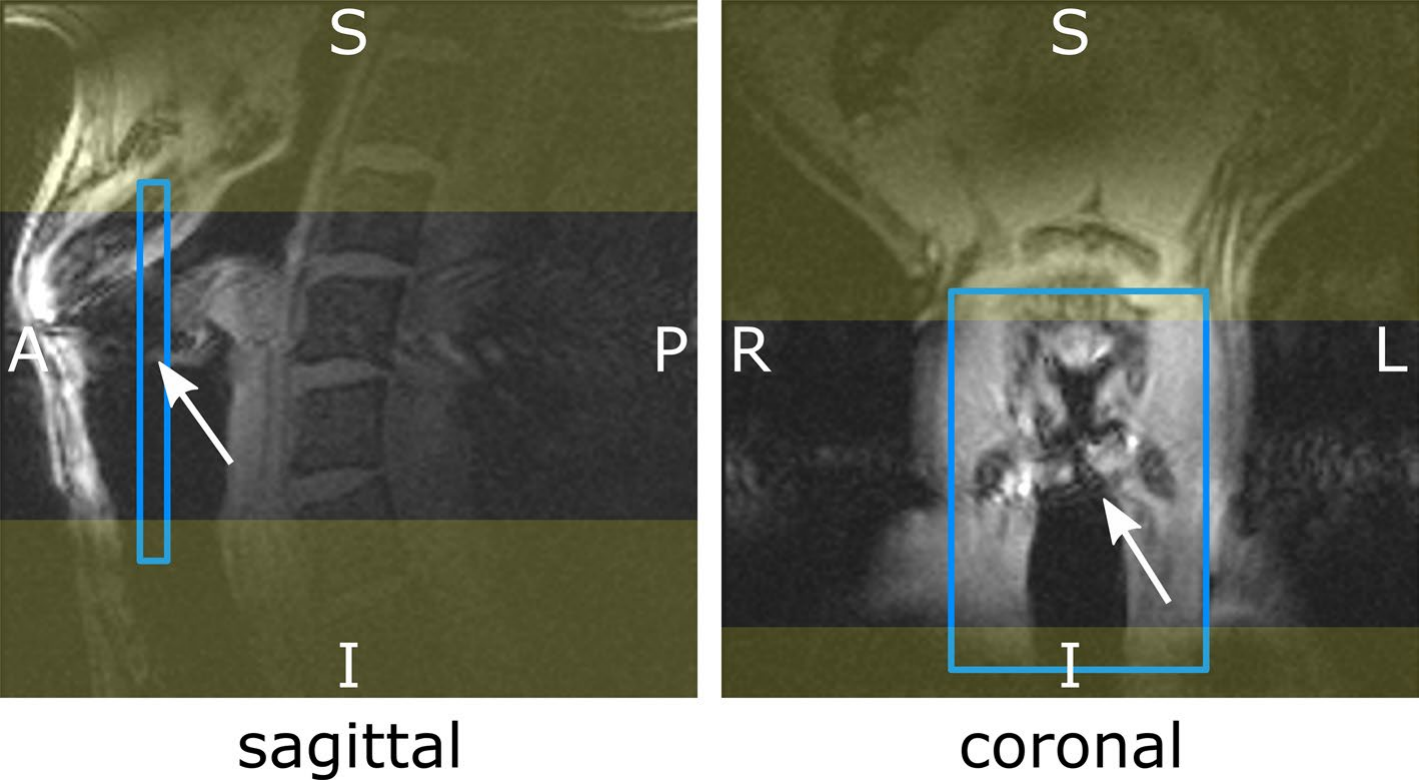
Gradient echo images acquired with a regular FLASH sequence during phonation. In the sagittal view, the position of the image slice for the SPIRE acquisition is indicated by the blue lines and in the coronal view the position and size of the field of view is shown. Yellow areas indicate the saturation bands applied during the SPIRE sequence. White arrows indicate the position of the oscillating vocal folds, which is also revealed by motion artifacts in the AP and RL direction. The slice position in AP direction is chosen halfway between the thyroid cartilage and the vocal process. In the RL direction, moderate aliasing of neck tissue is accepted to reduce measurement time

To avoid signals from above and below the imaging FOV in SI direction to fold over on the vocal folds, two saturation bands (width = 80 mm) were applied to minimize these signals (Fig. 2).

SPIRE image acquisitions cannot be performed within one continued phonation, as the total measurement time can be several minutes long. To detect and correct for breathing motion and involuntary laryngeal movements, 1D navigator measurements in both SI and LR direction were integrated into the SPIRE sequence and applied every 100 ms, together with the saturation bands (Fig. 1a).

### Simulation of 1D and 2D encoding schemes

**Fig. 3:**
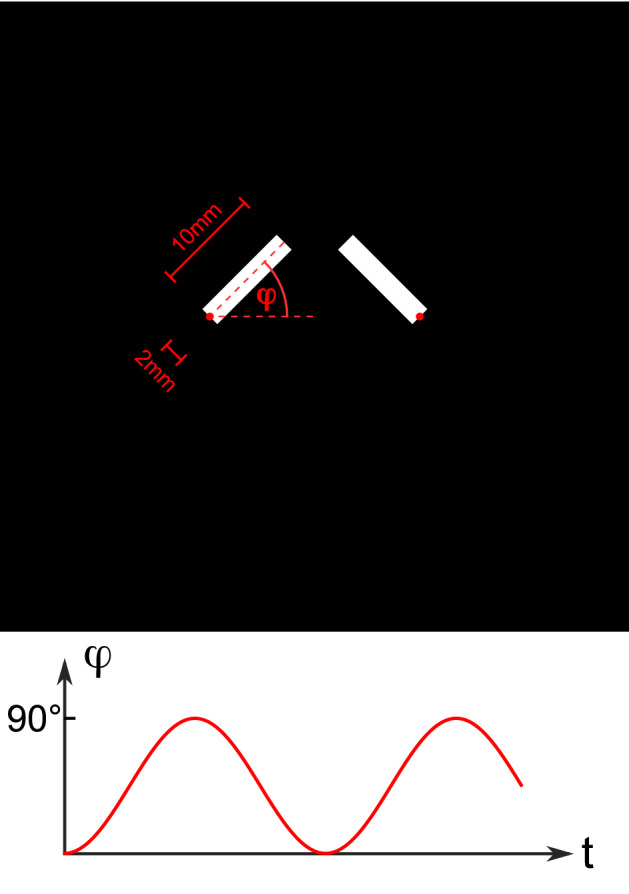
Schematic representation of the phantom and its motion. Two white bars with width 2 mm and length 10 mm rotate about their pivot points (red dots). The opening angle changes sinusoidally and is always the same on both sides

To demonstrate that the SPIRE sequence can encode 2D motion patterns, we simulated the signal acquisition with SPIRE and compared it to the previously presented FLASH sequence for fast 1D motion encoding [31]. A numerical motion phantom was designed consisting of two elongated rectangles that rotate by 90° about two pivot points at their ends (similar to a double-sided swing door) (Fig. 3). To simulate the MR signal, about 15 individual signal sources (isochromates) per pixel were placed within the initial boundaries of the rectangles, and a periodic in-plane motion path was defined for each isochromate. Simulations were performed with five different frequencies, chosen such that the fastest isochromate moves by ${N}_{\mathrm{px}}$ = 0, 1, 5, 25, or 50 pixels during phase encoding:

$${v}_{\mathrm{max}}={N}_{\mathrm{px}}\frac{\Delta x}{{t}_{\mathrm{PE}\ \mathrm{max}}}.$$

With the given measurement parameters, this translates to ${v}_{\mathrm{max}}=0, 1.4, 7.1, 35.5, 71.0\ \mathrm{ m}/\mathrm{s}$; the simulation with ${v}_{\mathrm{max}}=0\ \mathrm{m}/\mathrm{s}$ served as the reference. The signal evolution was then calculated for the SPIRE sequence with similar parameters as in the volunteer experiments, but a quadratic FOV = (60 mm)^2^ and 64^2^ matrix size. For the 1D motion encoding sequence, the same spatial resolution and maximum phase encoding time (660 µs) were chosen, and a readout bandwith of 1000 Hz/pixel was used. An analytical solution of the Bloch equations [42, 43] was used to calculate the evolution of the magnetization, and the signals from the isochromates were summed and assigned to the corresponding k-space positions. Gradient spoiling was simulated by nulling the transverse magnetisation prior to each RF excitation (i.e., perfect spoiling). The simulation was carried out for ${N}_{\mathrm{phs}}=$ 20 different motion phases with increasing phase of the motion at the peak of the PE gradient lobe

$$\varphi_{i} = i \cdot \frac{2\pi }{{N_{{{\text{phs}}}} }},\quad {\text{with}}\quad i = 0,1, \ldots ,18,19.$$

From the simulated k-space data, images were reconstructed using a conventional 2D Fourier transform. Simulations were implemented in MATLAB (The MathWorks Inc., Natick, MA).

### Volunteer experiment

To image the vocal fold oscillations, two untrained, male volunteers were instructed to sing continuously during the SPIRE measurement, and to interrupt singing only briefly for breathing whenever necessary. To achieve a constant singing fundamental frequency, the volunteers were provided with visual feedback via an audio spectrogram shown on a screen where the target frequency (*f*_singing_ = 140 Hz) was highlighted. An electroglottogramm (EGG) [44, 45] was acquired during the measurement to gate the MR data during reconstruction. The EGG system (EGG-D400, Laryngograph Ltd., London, UK) has two MR-conditional electrodes, which were placed on the volunteers’ skin to the left and right of the larynx. The EGG provides an electrical signal that is proportional to the contact area of the vocal folds, and from which frequency, phase and the relative amplitude of the oscillation can be extracted. To avoid sampling of the same motion phase in each TR, the singing frequency was chosen such that it was different from integer multiples of the repetition frequency:

$$f_{TR} = \frac{1}{TR} \ne m \cdot f_{{{\text{singing}}}} \quad {\text{with}}\quad m \in {\mathbb{N}}.$$

For MR signal reception, a loop coil (R = 3.5 cm, Siemens Healthcare, Erlangen, Germany) was placed at the level of the vocal folds on top of the electrodes. To synchronize EGG and MR data, an optical trigger signal from the MR systems electrical cabinet was converted into an electrical signal and sent to the EGG recorder at every excitation pulse. EGG and optical trigger were recorded using SPEAD (Version 4.2.2, Laryngograph Ltd, Walligton, UK) and exported using the two stereo channels in the WAV file format.

The extent of the neck in LR direction is usually much smaller compared to SI direction, but can vary between volunteers. Here, we use a rectangular field of view by increasing the step-size in k-space, while accepting small aliasing of the surrounding tissue without compromising the signal from the vocal fold tissue (Fig. 2).

To achieve an image resolution below 1 mm, a rectangular FOV of 60 mm × 41 mm was chosen using a matrix size of 64 × 44. With *α* = 10°, TE = 1.2 ms, TR = 2.45 ms and 24 repetitions, the total acquisition time was 2 min 58 s for a single 2D slice. With these parameters ${t}_{\mathrm{PE max}}$ was not limited by the scanner hardware, but by peripheral nerve stimulation (PNS) caused by the rapid gradient switching. To avoid PNS, slew rates were limited to 121 mT/m/ms (Fig. 1b).

### Image reconstruction

In a first step, for each measured k-space point the phase of the vocal fold oscillation cycle at the peak of the PE gradients was identified from the simultaneously acquired EGG data. Therefore, a sine function was fit to the EGG data to extract amplitude, fundamental frequency and phase of the oscillation. While the measured EGG signal is not sinusoidal in shape, its fundamental frequency still provides a suitable measure of the vocal fold oscillation frequency. The phase of the fitted EGG waveform was used to sort the acquired data into 10 different k-spaces to allow a fluid reconstruction of the motion without compromising SNR.

Prior to the image reconstruction, the shift in image space, *δx,* of each navigator $\tilde {I}_n\left(k\right)$ with respect to a reference navigator $\tilde I_{\mathrm{ref}}\left(k\right)$ was calculated. To estimate *δx*, we use the phase only cross correlation (POCC) which previously has been used to track passive markers during interventions [46, 47].

$$\mathrm{POCC}\left(k,n\right)=\frac{\tilde{I}_{\mathrm{ref}}{\left(k\right)}^{*}}{\Vert \tilde{I}_{\mathrm{ref}}\left(k\right)\Vert }\cdot \frac{\tilde{I}_{n}\left(k\right)}{\Vert \tilde{I}_{n}\left(k\right)\Vert }=\frac{{\tilde{I}_{\mathrm{ref}}\left(k\right)}^{*}}{\Vert \tilde{I}_{\mathrm{ref}}\left(k\right)\Vert }\cdot \frac{\tilde{I}_{\mathrm{ref}}\left(k\right){\mathrm{e}}^{-i\left(k\cdot \delta x\right)}}{\Vert \tilde{I}_{\mathrm{ref}}\left(k\right){\mathrm{e}}^{-i\left(k\cdot \delta x\right)}\Vert }={\mathrm{e}}^{-i\left(k\cdot \delta x\right)}.$$

As navigator data were only measured for every 40th k-space point, the shift was linearly interpolated for every SPI acquisition, and the shifts were corrected in k-space using the inverse Fourier shift theorem. A navigator acquired during the middle of the measurement was chosen as reference when the volunteer was singing with stable phonation. SPI data were rejected for image reconstruction when a shift of more than 3 mm in SI or RL direction was detected by the POCC—typically, these large shifts were associated with breathing. The additional fit-parameters (frequency, amplitude, *R*^2^) were used to detect the absence of phonation (during breathing) in the EGG signal and such data were also rejected.

Due to the retrospective gating, some k-space points were acquired multiple times and some were missing. To avoid undersampling artifacts, an iterative reconstruction with a total variation constraint [48, 49] along the temporal dimension was applied using the Berkeley Advanced Reconstruction Toolbox (BART, version 0.6.00), an open-source image-reconstruction framework for MRI [50]. Processing of MR and EGG data was implemented in MATLAB.

## Results

**Fig. 4:**
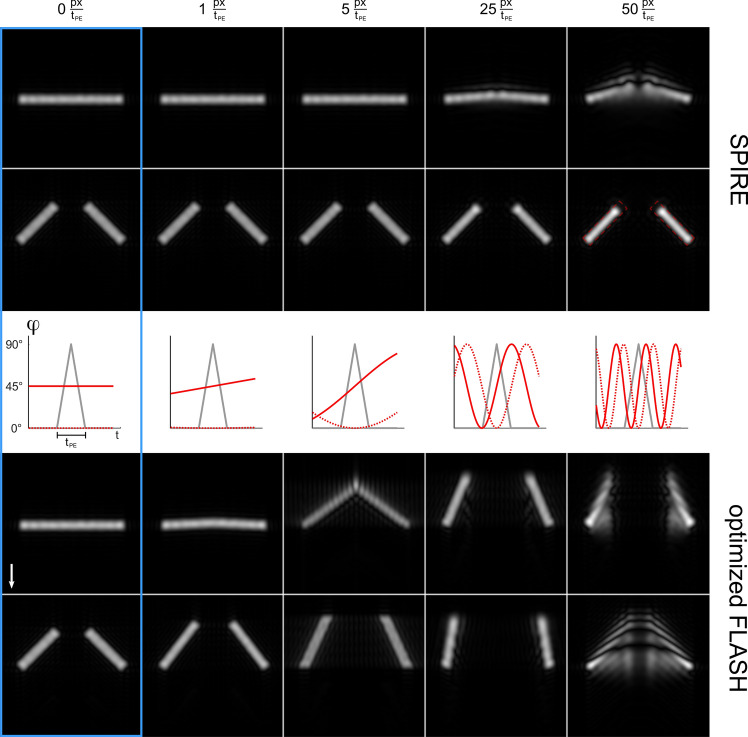
Reconstructed simulations of the door phantom using the SPIRE sequence (top) and optimized FLASH (bottom) for two distinct motion phases and five different velocities. Graphs in the center row show the behaviour of $\varphi (t)$ during the longest phase encoding gradient lobe (gray). Images inside the blue rectangle were simulated with a stationary phantom and thus serve as a ground truth. The arrow in the FLASH images indicates the direction of frequency encoding. Red continuous line: $\varphi =45^\circ$ at peak of *G*_PE_. Red dotted line: $\varphi =0^\circ$ at peak of *G*_PE_. The dashed line in the SPIRE image for *v* = 50 px/*t*_PE_ represents the phantom shape of the ground truth. The images show a central cutout from the phantom in Fig. 3

Figure 4 compares the simulated images of the numerical phantom at two different motion phases: at rest ($\varphi =0^\circ$), and at the highest angular velocity ($\varphi =45^\circ$). An animated version is provided in the supplementary material (Online Resource 1). In the optimized FLASH images artifacts and incorrect spatial encoding can be seen already for the lowest velocity of 1 px/*t*_PE_ which are a consequence of the delay between phase and frequency encoding. In addition, distortions of the geometrical shape are seen that increase with the motion velocity—for the highest velocity of 50 px/*t*_PE_ the signal is blurred over large areas and the geometry of the phantom cannot be recognized. With SPIRE, image information is always encoded at the correct position, only minor blurring and displacement artifacts become apparent at 25 px/*t*_PE_, and they are more pronounced around $\varphi =0^\circ$.

**Fig. 5:**
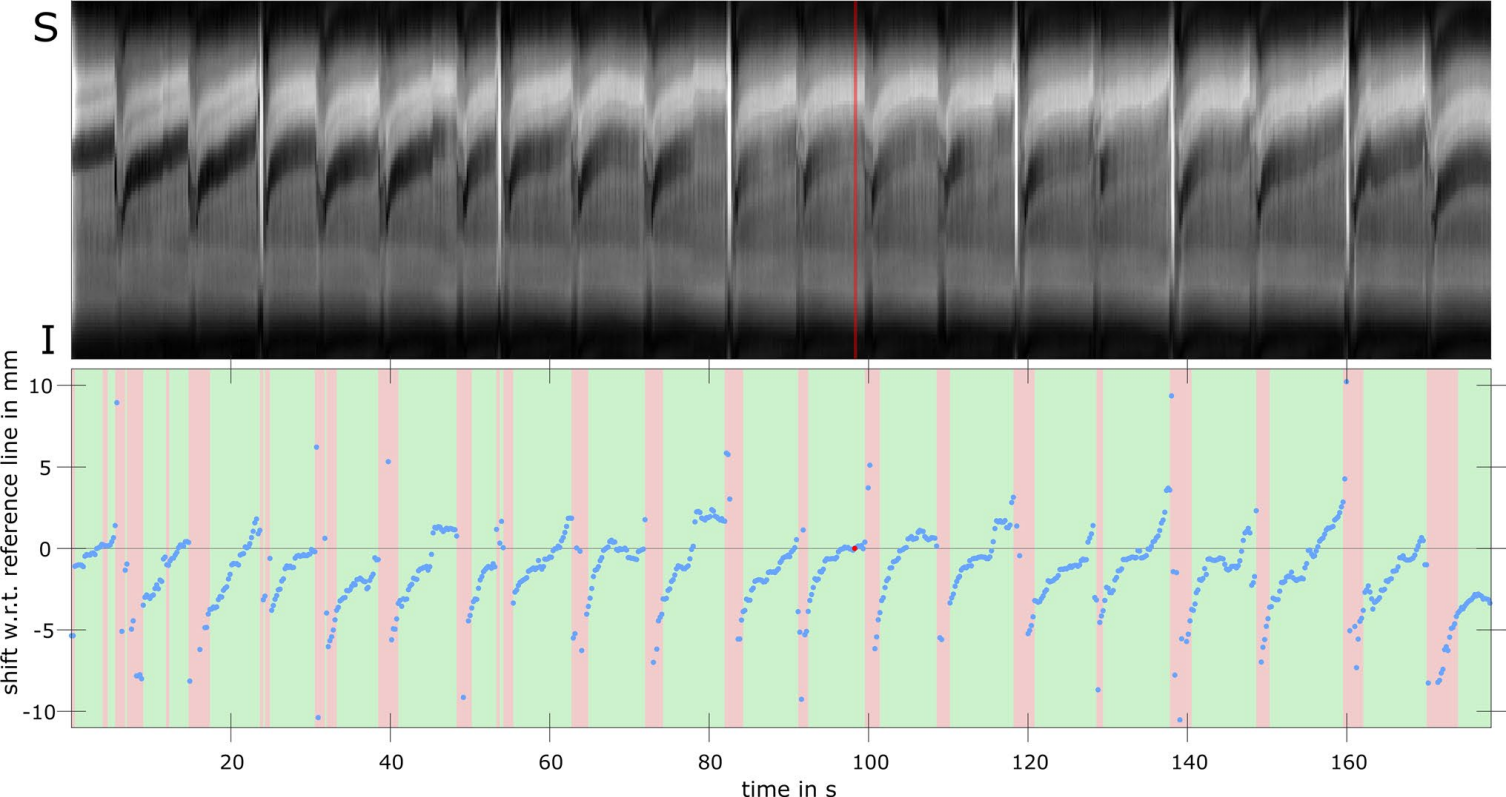
Top: All 852 SI-navigators acquired during the measurement, of which the 471st is taken as a reference line (shown in red). Bottom: blue points show the shift of each navigator with respect to the reference (red dot). A red background indicates those regions where data were rejected during reconstruction, because the shift was estimated to be larger than 3 mm, most likely due to the volunteer breathing

**Fig. 6:**
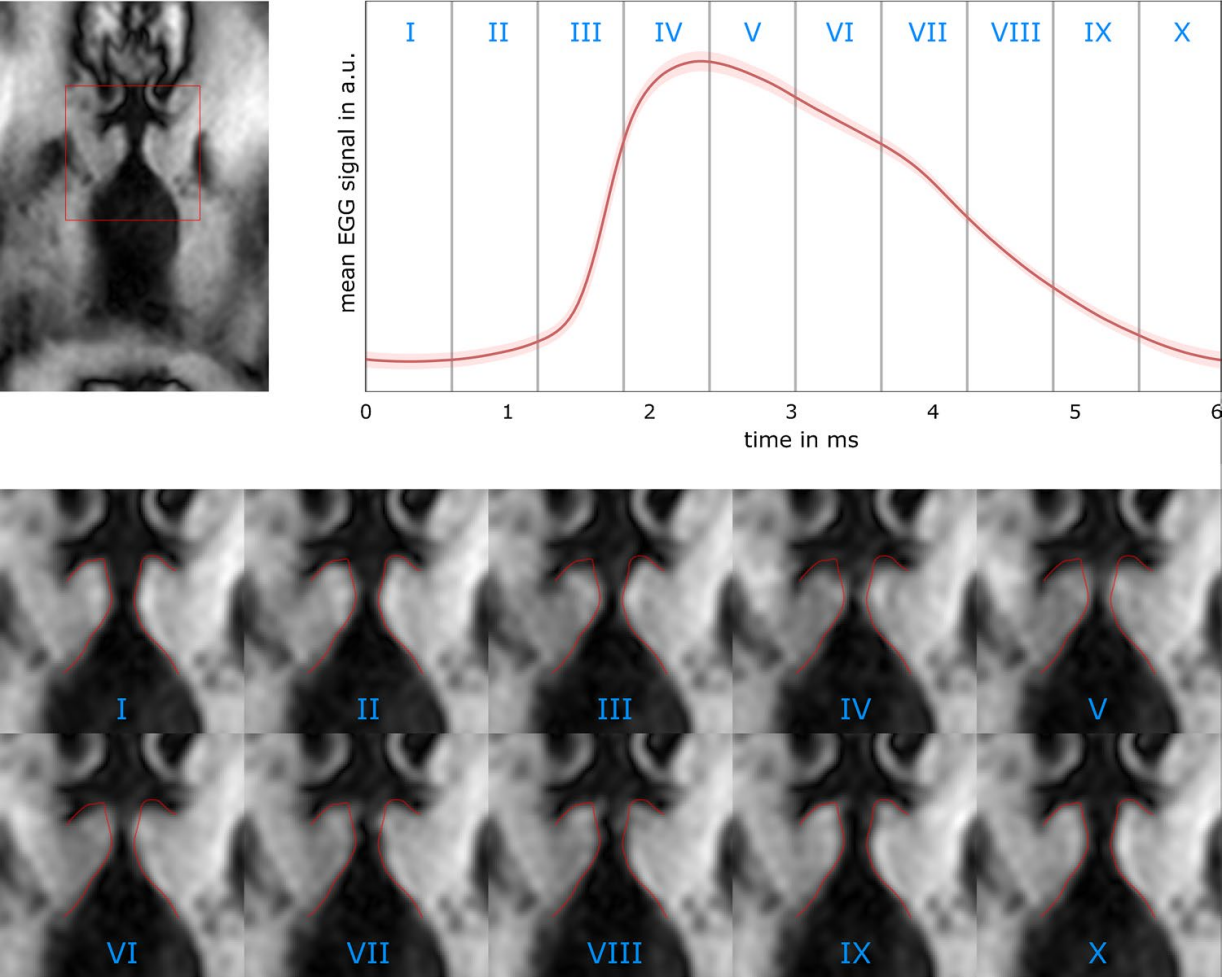
Individual frames reconstructed from measurements with volunteer one. Top: left: first frame of the reconstructed motion. The red square indicates the position of the ROI in the images below. Right: the red line shows the mean of the aligned EGG signals during all accepted SPI acquisitions, the red area indicates the standard deviation. Numbers in the plot relate the phase in the EGG cycle to the reconstructed image frame. All MR data where the peak of the PE gradients occurred within the first EGG bin are sorted into the first frame, etc. Bottom: ten frames of the reconstructed oscillation with a temporal resolution of Δ*t* = 606 µs per frame. The red line shows the shape of the vocal folds in frame 1 and is copied to all frames for comparison

Figure 5 shows all navigators acquired in SI direction as well as the calculated shift w.r.t. to the empirically chosen reference line (here: line 471). Between the short breathing interruptions, the position of the vocal fold can be clearly identified, and a slow upward movement can be observed. About 28% of the acquired data is rejected mainly because of a lack of phonation as detected by the EGG or uncorrectable larynx shifts in the navigators. For the accepted SPI data, the mean phonation frequency was 165.1 $\pm$ 3.4 Hz (Online Resource 2). Due to data rejection, 48,365 SPI acquisitions were accepted that were sorted into 10 dynamic 2D k-space data sets (temporal resolution: 606 µs) with 64 × 44 data points each resulting in 17% empty k-space points. In the second volunteer, the mean frequency was lower (148.3 $\pm$ 4.5 Hz) and about 34% of the acquired MR data were rejected (24% of k-space remained empty). Figure 6 shows each dynamic frame and the averaged EGG signal from all acquisitions over one oscillation cycle. In frames 1 and 2, the low EGG signal is consistent with an open state of the vocal folds, and in frame 3 the closing of the vocal folds begins. This is reflected in the SPIRE images, as in the third frame the lower parts of both vocal folds move toward the center of the glottis (the opening between the vocal folds). In frames 4 and 5, the vocal folds are fully closed and the contact point between both the left and right vocal fold moves upward. In the following frames, the vocal folds open again: in frame 6, the lower parts start to move back to the side, and in frame 7 a kink in the EGG signal is seen which marks the beginning of the separation of the upper parts of the vocal folds [51–53] that can also be seen in the MR images 7–10. At the contact point between both the vocal folds, the SPIRE signal is low compared to the rest of the tissue, making it hard to define the contact area. A video of the vocal fold oscillation slowed down 100 times can be found in the supplementary material (Online Resource 3). Minor signal oscillations at the image edges in LR direction stem from the EGG electrodes vibrating on the skin, excited by the vibration in the larynx. A comparison of the image reconstruction without prior shift correction is shown in Online Resource 4. The results for the second volunteer can be found in the supplementary material (Online Resource 5 and 6). Due to the lower singing frequency, the temporal resolution of the reconstructed images is 675 µs.

## Discussion and conclusions

In this pilot study, for the first time, dynamic 2D MR images of the oscillating vocal folds during phonation are presented. The dynamic image information correlates well with the oscillation phases from the EGG-signal, and the open and closed phase can be clearly distinguished in the SPIRE images.

The simulations of the SPIRE signals show that a 2D SPI method is needed to overcome the image artifacts caused by the fast 2D oscillations, and that the previously presented 1D method cannot be applied in this case. In the simulated phantom images, artifacts are more pronounced in stationary phases of the motion ($\varphi =0^\circ )$ than at maximum velocity ($\varphi =45^\circ )$. This counterintuitive result is a consequence of the symmetry of the motion pattern: for small velocities, each isochromate effectively performs a linear motion over *t*_PE_ and a mean position is encoded. For higher velocities, motion can no longer be linearly approximated and the sequence becomes motion sensitive. However, if the motion pattern has odd symmetry with respect to the center of the PE gradient, the encoded phase due to motion is rewound over the phase encoding gradient and only the mean position is encoded. This is the case at $\varphi =45^\circ$, where a uniform distortion of the object shape can be seen in the images simulated with *v* = 50 px/*t*_PE_: The two bars appear shorter, because the mean position of an object moving along an arc is not located on the same arc but on a smaller radius and the object appears smaller (see second row in Fig. 4).

Even though the expected velocity of the mucosal wave is about $4\ \mathrm{m}/\mathrm{s}=2.8\ \frac{\Delta x}{{t}_{\mathrm{PE}}}$, the correct representation of the shape of the vocal folds is important to understand the motion dynamics, so that the use of the more time-consuming SPI method is warranted. Single-point imaging has previously been used for high-speed acquisition, where rapidly switched currents were measured limited only by the bandwidth of the ADC [61]. A similar approach for sub-millisecond temporal resolution was recently presented for dynamic imaging of aortic heart valves [60], where short gradient pulses were used in readout direction.

The use of navigators allows to compensate for position differences between phonation cycles, and preserves edges in the non-oscillation tissues. In general, the applied POCC algorithm can correct in-plane movement, but is not suitable for out-of-slice motion, which might occur when larynx motion and slice orientation are not parallel. Future work will focus on reproducible positioning of the volunteers larynx and improvements in navigator signal to better preclude and correct subject motion. Additionally, prior knowledge about the surrounding tissue might improve image reconstruction.

A limitation of SPIRE is that with the current contrast the contact area between the left and right vocal folds cannot be clearly identified. This low signal intensity is most likely caused by the MRI properties of the epithelial tissue—as the contact area cannot be measured precisely, the dynamics of the mucosal wave is hard to identify. This limitation might be overcome by using image contrasts the enhance liquid-containing tissues; for example, a balanced steady state free precession (bSSFP) technique might be suitable, which can easily be integrated into the sequence by rewinding the gradient moments between each RF excitation.

At the spatial resolution used in this work, a temporal resolution of 500 µs can theoretically be achieved with the available gradient system. However, currently PNS limits prohibit the use of ultrafast gradient switching, and thus a sub-optimal TR had to be used which comes at the expense of increased measurement time or fewer acquired data points.

In the case of patients with vocal fold pathologies, repeated phonation for 3 min might be strenuous and improvements to acquisition times are needed. In this work we have not explored the possibility of parallel imaging. Accelerated 3D image acquisitions with a 2D Poisson-disc undersampling pattern in phase and partition encoding direction have been shown to work well with regularized reconstructions [54–56]. In this work, however, acquisitions using a twofold undersampled poisson disc and reconstructed using an additional l1-wavelet regularization along spatial image dimensions could not reach the image clarity visible in the reconstructions where all k-space points are acquired even if acquisition time was kept the same (intentional). We assume that l1-wavelet regularization does not result in good sparsity in small matrix sizes.

An interesting application for SPIRE is the analysis of different vocal registers in the human voice. The vibratory motion of the vocal folds can change its quality to provide the wide range of frequencies. While in the modal register (usual register for speech and singing) oscillations involve body and cover of the vocal folds, in the falsetto register (used for higher frequencies) only the thin cover layer vibrates and the body remains almost motionless. SPIRE 2D imaging of these registers could significantly improve our understanding of voice production as tissue motion below the closed vocal folds can be visualized, which cannot be seen from a top-down perspective as in most optical examinations. SPIRE could also be used in phonosurgery [57, 58] to study the perturbation of the vibratory movement by vocal fold lesions in pre- and post-surgical analysis. Additionally, SPIRE enables to study the vocal fold oscillation in subjects where the top-down view of the oscillating vocal folds is blocked, e.g., by verticular fold hyperadduction or oscillation. This can be the case in muscle tension dysphonia [59] or special vocal techniques used in contemporary musical style singing like rattle [62]. Beyond vocal fold imaging, SPIRE could be applied to any periodic two-dimensional in-plane motion, as long as a gating signal with high temporal resolution is available.

## Supplementary Information

Below is the link to the electronic supplementary material.

Supplementary file1 (AVI 3539 KB)

Supplementary file2 (TIF 1498 KB)

Supplementary file3 (AVI 2836 KB)

Supplementary file4 (AVI 547 KB)

Supplementary file5 (AVI 2973 KB)

Supplementary file6 (TIF 91568 KB)

Supplementary file7 (DOCX 6286 KB)

## References

[1] Echternach M, Döllinger M, Sundberg J, Traser L, Richter B (2013). Vocal fold vibrations at high soprano fundamental frequencies. J Acoust Soc Am.

[2] Naunheim MR, Carroll TL (2017). Benign vocal fold lesions: update on nomenclature, cause, diagnosis, and treatment. Curr Opin Otolaryngol Head Neck Surg.

[3] Echternach M, Burk F, Burdumy M, Herbst CT, Köberlein M, Döllinger M, Richter B (2017). The influence of vocal fold mass lesions on the passaggio region of professional singers. Laryngoscope.

[4] Yonekawa H (1988). A clinical study of Reinke’s edema. Auris Nasus Larynx.

[5] Watanabe T, Kaneko K, Sakaguchi K, Takahashi H (2016). Vocal-fold vibration of patients with Reinke’s edema observed using high-speed digital imaging. Auris Nasus Larynx.

[6] Giovanni A, Chanteret C, Lagier A (2007). Sulcus vocalis: a review. Eur Arch Otorhinolaryngol.

[7] Schultz P (2011). Vocal fold cancer. Eur Ann Otorhinolaryngol Head Neck Dis.

[8] Marioni G, Marchese-Ragona R, Cartei G, Marchese F, Staffieri A (2006). Current opinion in diagnosis and treatment of laryngeal carcinoma. Cancer Treat Rev.

[9] van Balkum M, Buijs B, Donselaar EJ, Erkelens DCA, Fernandes EGL, Wegner I, Grolman W, Janssen LM (2017). Systematic review of the diagnostic value of laryngeal stroboscopy in excluding early glottic carcinoma. Clin Otolaryngol.

[10] Paul BC, Chen S, Sridharan S, Fang Y, Amin MR, Branski RC (2013). Diagnostic accuracy of history, laryngoscopy, and stroboscopy. Laryngoscope.

[11] Titze IR (1994). Principles of voice production.

[12] Hirano M (1974). Morphological structure of the vocal cord as a vibrator and its variations. Folia Phoniatr (Basel).

[13] Hirano M (1988). Vocal mechanisms in singing: laryngological and phoniatric aspects. J Voice.

[14] Vahabzadeh-Hagh AM, Zhang Z, Chhetri DK (2018). Hirano’s cover-body model and its unique laryngeal postures revisited. Laryngoscope.

[15] Shau YW, Wang CL, Hsieh FJ, Hsiao TY (2001). Noninvasive assessment of vocal fold mucosal wave velocity using color Doppler imaging. Ultrasound Med Biol.

[16] Oertel M (1895). Das laryngo-stroboskop und die laryngo-stroboskopische untersuchung. Arch Laryngol Rhinol.

[17] Mehta DD, Hillman RE (2012). Current role of stroboscopy in laryngeal imaging. Curr Opin Otolaryngol Head Neck Surg.

[18] Olthoff A, Woywod C, Kruse E (2007). Stroboscopy versus high-speed glottography: a comparative study. Laryngoscope.

[19] de Mul FFM, George NA, Qiu Q, Rakhorst G, Schutte HK (2009). Depth-kymography of vocal fold vibrations: part II. Simulations and direct comparisons with 3D profile measurements. Phys Med Biol.

[20] Coughlan CA, Chou L, Jing JC, Chen JJ, Rangarajan S, Chang TH, Sharma GK, Cho K, Lee D, Goddard JA, Chen Z, Wong BJF (2016). In vivo cross-sectional imaging of the phonating larynx using long-range Doppler optical coherence tomography. Sci Rep.

[21] Jing B, Ge Z, Wu L, Wang S, Wan M (2018). Visualizing the mechanical wave of vocal fold tissue during phonation using electroglottogram-triggered ultrasonography. J Acoust Soc Am.

[22] Döllinger M, Kobler J, Berry DA, Mehta DD, Luegmair G, Bohr C (2011). Experiments on analysing voice production: excised (human, animal) and in vivo (animal) approaches. Curr Bioinform.

[23] Herbst CT, Hampala V, Garcia M, Hofer R, Svec JG (2017). Hemi-laryngeal setup for studying vocal fold vibration in three dimensions. J Vis Exp.

[24] Echternach M, Markl M, Richter B (2012). Dynamic real-time magnetic resonance imaging for the analysis of voice physiology. Curr Opin Otolaryngol Head Neck Surg.

[25] Scott AD, Wylezinska M, Birch MJ, Miquel ME (2014). Speech MRI: morphology and function. Phys Med.

[26] Lingala SG, Sutton BP, Miquel ME, Nayak KS (2016). Recommendations for real-time speech MRI. J Magn Reson Imaging.

[27] Burdumy M, Traser L, Richter B, Echternach M, Korvink JG, Hennig J, Zaitsev M (2015). Acceleration of MRI of the vocal tract provides additional insight into articulator modifications. J Magn Reson Imaging.

[28] Echternach M, Burk F, Burdumy M, Traser L, Richter B (2016). Morphometric differences of vocal tract articulators in different loudness conditions in singing. PLoS ONE.

[29] Burdumy M, Traser L, Burk F, Richter B, Echternach M, Korvink JG, Hennig J, Zaitsev M (2017). One-second MRI of a three-dimensional vocal tract to measure dynamic articulator modifications. J Magn Reson Imaging.

[30] Zhao Z, Lim Y, Byrd D, Narayanan S, Nayak KS (2021). Improved 3D real-time MRI of speech production. Magn Reson Med.

[31] Fischer J, Abels T, Özen AC, Echternach M, Richter B, Bock M (2020). Magnetic resonance imaging of the vocal fold oscillations with sub-millisecond temporal resolution. Magn Reson Med.

[32] Özen AC, Traser L, Echternach M, Dadakova T, Burdumy M, Richter B, Bock M (2016). Ensuring safety and functionality of electroglottography measurements during dynamic pulmonary MRI. Magn Reson Med.

[33] Emid S, Creyghton JHN (1985). High resolution NMR imaging in solids. Phys BC.

[34] Weiger M, Pruessmann KP (2019). Short-T2 MRI: principles and recent advances. Prog Nucl Magn Reson Spectrosc.

[35] Muir CE, Balcom BJ (2013). A comparison of magnetic resonance imaging methods for fluid content imaging in porous media. Magn Reson Chem.

[36] Marica F, Goora FG, Balcom BJ (2014). FID-SPI pulse sequence for quantitative MRI of fluids in porous media. J Magn Reson.

[37] Fang Z, Hoepfel D, Winter K (2001). Application of single point imaging (SPI) to solid state materials. Magn Reson Imaging.

[38] Özen AC, Ludwig U, Öhrström LM, Rühli FJ, Bock M (2016). Comparison of ultrashort echo time sequences for MRI of an ancient mummified human hand: comparison of UTE sequences for mummy MRI. Magn Reson Med.

[39] Balcom BJ, Macgregor RP, Beyea SD, Green DP, Armstrong RL, Bremner TW (1996). Single-point ramped imaging withT1Enhancement (SPRITE). J Magn Reson A.

[40] Froidevaux R, Weiger M, Rösler MB, Brunner DO, Pruessmann KP (2021). HYFI: hybrid filling of the dead-time gap for faster zero echo time imaging. NMR Biomed.

[41] Grodzki DM, Jakob PM, Heismann B (2012). Ultrashort echo time imaging using pointwise encoding time reduction with radial acquisition (PETRA). Magn Reson Med.

[42] Torrey HC (1949). Transient nutations in nuclear magnetic resonance. Phys Rev.

[43] Morris GA, Chilvers PB (1994). General analytical solutions of the Bloch equations. J Magn Reson A.

[44] Fourcin AJ, Abberton E (1971). First applications of a new laryngograph. Med Biol Illus.

[45] Herbst CT (2020). Electroglottography—an update. J Voice.

[46] de Oliveira A, Rauschenberg J, Beyersdorff D, Semmler W, Bock M (2008). Automatic passive tracking of an endorectal prostate biopsy device using phase-only cross-correlation. Magn Reson Med.

[47] Reichert A, Bock M, Reiss S, Overduin CG, Fütterer JJ, Krafft AJ (2018). Simultaneous slice excitation for accelerated passive marker tracking via phase-only cross correlation (POCC) in MR-guided needle interventions. Magn Reson Mater Phys Biol Med.

[48] Rudin LI, Osher S, Fatemi E (1992). Nonlinear total variation based noise removal algorithms. Phys Nonlinear Phenom.

[49] Block KT, Uecker M, Frahm J (2007). Undersampled radial MRI with multiple coils. Iterative image reconstruction using a total variation constraint. Magn Reson Med.

[50] Uecker M, Ong F, Tamir JI, Bahri D, Virtue P, Cheng JY, Zhang T, Lustig M (2015) Berkeley advanced reconstruction toolbox. In: Proceedings of the international society for magnetic resonance in medicine, Toronto, p 2486

[51] Berke GS, Moore DM, Hantke DR, Hanson DG, Gerratt BR, Burstein F (1987). Laryngeal modeling: theoretical, in vitro, in vivo. Laryngoscope.

[52] Baken RJ, Orlikoff RF (2000). Clinical measurement of speech and voice.

[53] Hampala V, Garcia M, Švec JG, Scherer RC, Herbst CT (2016). Relationship between the electroglottographic signal and vocal fold contact area. J Voice.

[54] Lustig M, Donoho D, Pauly JM (2007). Sparse MRI: the application of compressed sensing for rapid MR imaging. Magn Reson Med.

[55] Moghari MH, Uecker M, Roujol S, Sabbagh M, Geva T, Powell AJ (2018). Accelerated whole-heart MR angiography using a variable-density poisson-disc undersampling pattern and compressed sensing reconstruction. Magn Reson Med.

[56] Cristobal-Huerta A, Poot DHJ, Vogel MW, Krestin GP, Hernandez-Tamames JA (2019). Compressed sensing 3D-GRASE for faster high-resolution MRI. Magn Reson Med.

[57] Remacle M, Friedrich G, Dikkers F, de Jong F (2003). Phonosurgery of the vocal folds: a classification proposal. Eur Arch Otorhinolaryngol.

[58] Chiesa-Estomba CM, González-García JA, Larruscain E, Calvo-Henríquez C, Mayo-Yáñez M, Sistiaga-Suarez JA (2019). CO_2_ transoral laser microsurgery in benign, premalignant and malignant (Tis, T1, T2) lesion of the glottis. A literature review. Medicines.

[59] Stager SV, Bielamowicz SA, Regnell JR, Ashmit G, Barkmeier JM (2000). Supraglottic activity. J Speech Lang Hear Res.

[60] Zhong Z, Sun K, Karaman MM, Zhou XJ (2021). Magnetic resonance imaging with submillisecond temporal resolution. Magn Reson Med.

[61] Bakker CJG, van Gorp JS, Verwoerd JL, Westra AH, Bouwman JG, Zijlstra F, Seevinck PR (2013). Multiple single-point imaging (mSPI) as a tool for capturing and characterizing MR signals and repetitive signal disturbances with high temporal resolution: the MRI scanner as a high-speed camera. Magn Reson Imaging.

[62] Aaen M, McGlashan J, Sadolin C (2020). Laryngostroboscopic exploration of rough vocal effects in singing and their statistical recognizability: an anatomical and physiological description and visual recognizability study of distortion, growl, rattle, and grunt using laryngostroboscopic imaging and panel assessment. J Voice.

