# Supplementary figures and images for: Sub-millisecond 2D MRI of the vocal fold oscillation using single-point imaging with rapid encoding
Source: MAGMA. 2021 Sep 20;35(2):301–10. doi: 10.1007/s10334-021-00959-4 (PMC8995286; doi:10.1007/s10334-021-00959-4)

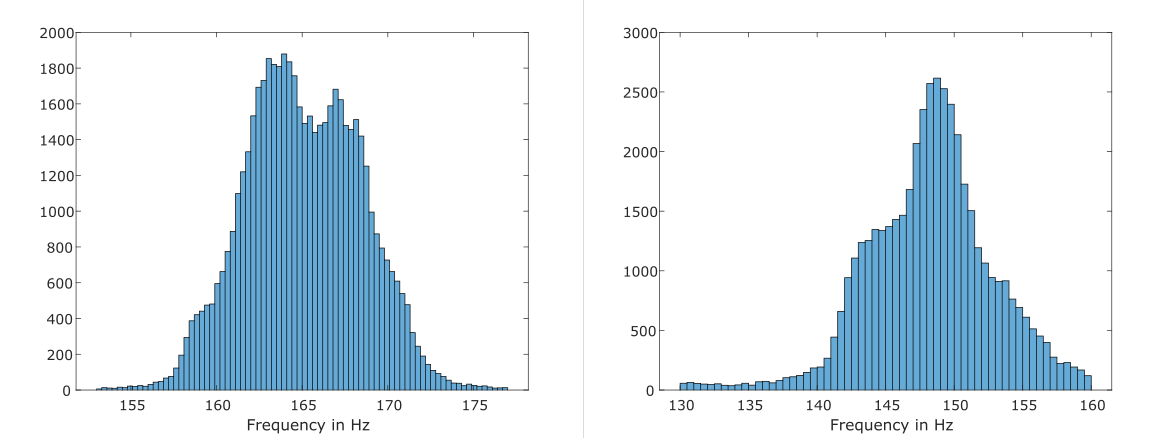

Supplement: Supplementary file 2 — Supplementary file2 (TIF 1498 KB) [file 10334_2021_959_MOESM2_ESM.tif]
