# Supplementary material for: Sub-millisecond 2D MRI of the vocal fold oscillation using single-point imaging with rapid encoding
Source: MAGMA. 2021 Sep 20;35(2):301–10. doi: 10.1007/s10334-021-00959-4 (PMC8995286; doi:10.1007/s10334-021-00959-4)
Supplement: Supplementary file 7 — Supplementary file7 (DOCX 6286 KB) [file 10334_2021_959_MOESM7_ESM.docx]

Online Resource 1: Animation of reconstructed simulations with five different peak velocities (from left to right: 0, 1, 5, 25, 50 px/t_PE_). Top row: reconstruction of SPIRE simulation. Bottom row: reconstruction of optimized FLASH sequence


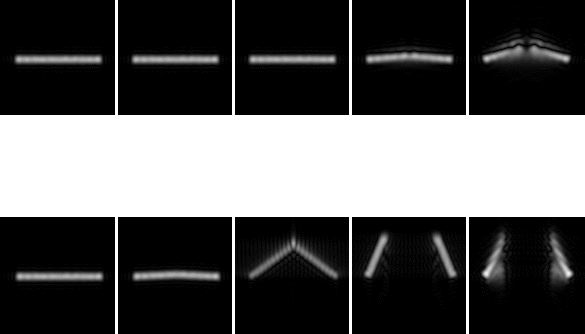


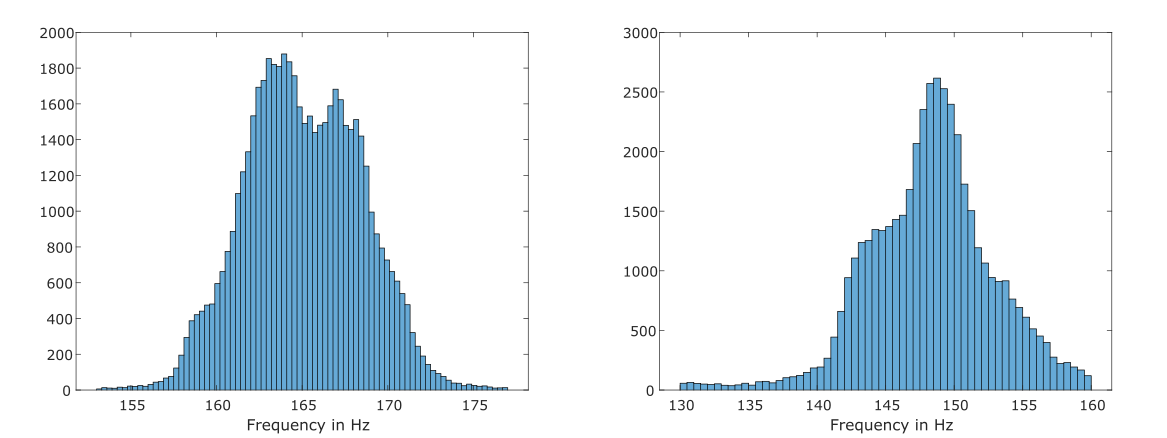


Online Resource 2: Histrogram of the phonation frequencies during each sequence block for volunteer one (left) and volunteer two (right).


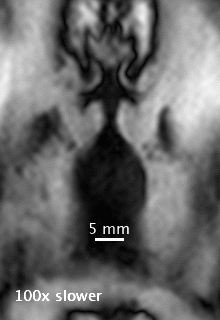


Online Resource 3: Animation of the reconstructed vocal folds oscillation measurement with volunteer one. Each of the 10 frames has a temporal resolution of 606μs and the motion is replayed at 1% of its original speed.


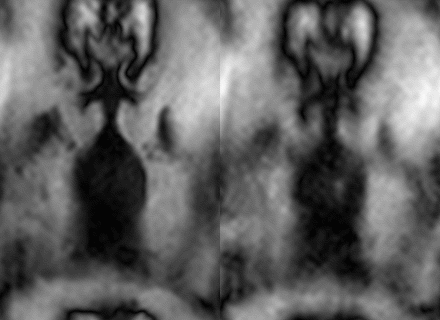


Online Resource 4: Animated comparison of reconstruction with (left) and without (right) correction for LR and SI motion of the larynx.


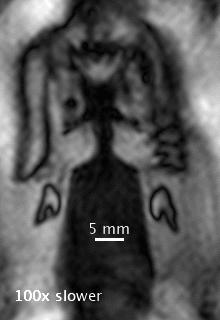


Online Resource 5: Animation of the measurement with volunteer two. Each of the 10 frames has a temporal resolution of 675μs and the motion is replayed at 1% of its original speed.


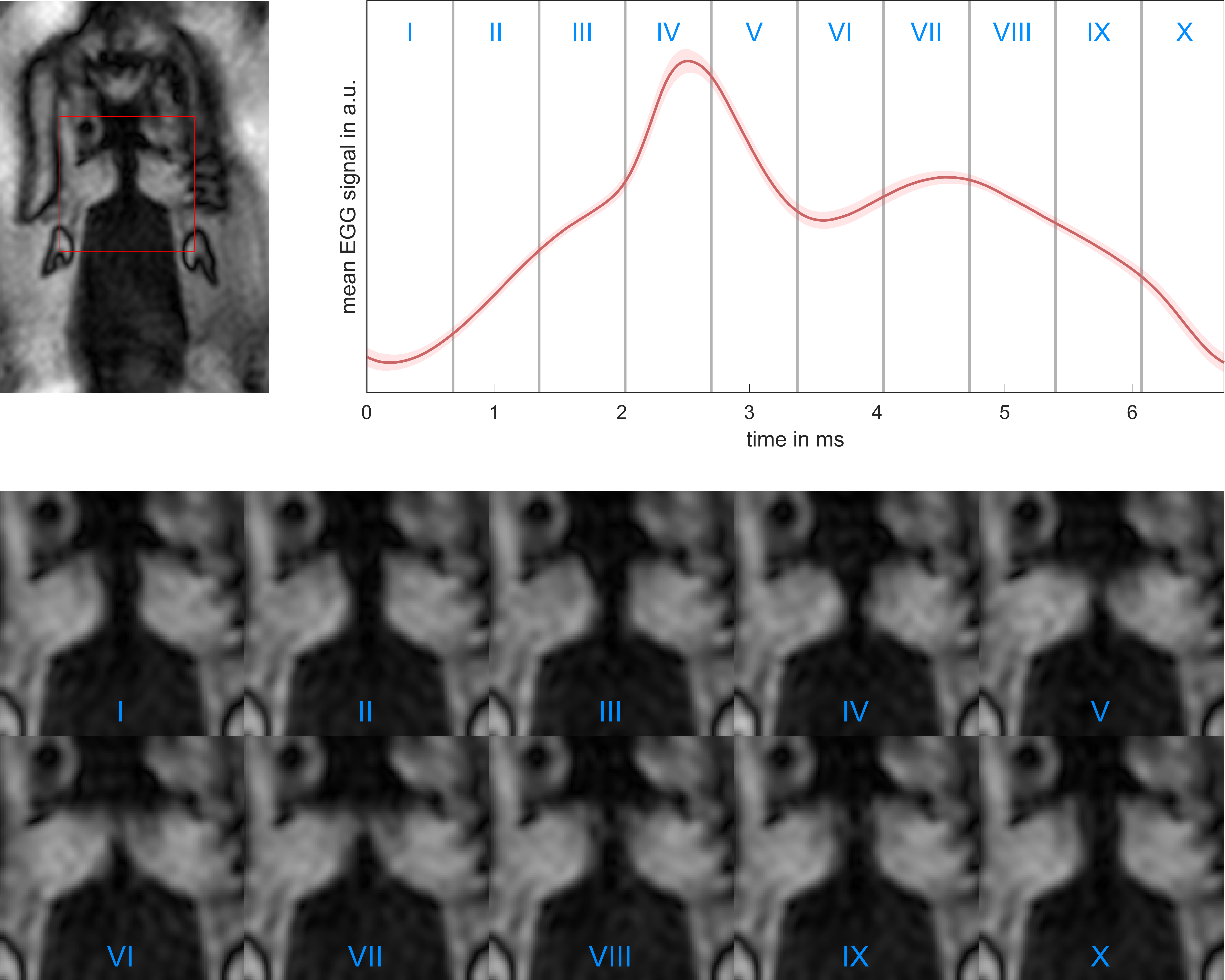


Online Resource 6: Individual frames reconstructed from measurements with volunteer two. Top: Left: First frame of the reconstructed motion. The red square indicates the position of the ROI in the images below. Right: The red line shows the mean of the aligned EGG signals during all accepted SPI acquisitions, the red area indicates the standard deviation. Numbers in the plot relate the phase in the EGG cycle to the reconstructed image frame. All MR-data where the peak of the PE gradients occurred within the 1^st^ EGG-bin are sorted into 1^st^ frame, etc. Bottom: 10 frames of the reconstructed oscillation with a temporal resolution of Δt = 675 μs per frame. The red line shows the shape of the vocal folds in frame 1 and is copied to all frames for comparison.
